# Allergic Bronchopulmonary Aspergillosis in a Patient With Poorly Controlled Bronchial Asthma

**DOI:** 10.7759/cureus.46666

**Published:** 2023-10-08

**Authors:** Dimithra R Gamalathge, Solangaratchige Don Harshana Sameera Perera

**Affiliations:** 1 Internal Medicine, Colombo East Base Hospital, Colombo, LKA; 2 Internal Medicine, National Hospital of Sri Lanka, Colombo, LKA

**Keywords:** cystic fibrosis, allergic bronchopulmonary aspergillosis (abpa), immunoglobulin ige, uncontrolled asthma, poor asthma outcomes

## Abstract

Allergic bronchopulmonary aspergillosis (ABPA) is a notable complication in patients with chronic lung diseases, such as chronic bronchial asthma and cystic fibrosis, presenting challenges in diagnosis and management. ABPA is an allergic response to multiple antigens expressed by *Aspergillus fumigatus* in the lung mucosa, resulting in airway inflammation and damage. This study discusses a 58-year-old male patient with a history of longstanding bronchial asthma for 28 years who presented with worsening respiratory symptoms.

The patient’s blood investigations showed peripheral eosinophilia, increased total serum immunoglobulin IgE, and positive Aspergillus serology. Bronchoalveolar lavage samples showed a significant increase in Aspergillus antigens, along with positive radiological findings, leading to the diagnosis of ABPA. He was successfully treated with a combination of dual antifungal therapy, systemic corticosteroids, inhaled corticosteroids, and bronchodilators. This study emphasizes the importance of considering ABPA in patients with chronic bronchial asthma experiencing deteriorating respiratory symptoms and highlights the significance of a multidisciplinary approach for accurate diagnosis and effective management of this condition.

## Introduction

Allergic bronchopulmonary aspergillosis (ABPA) predominantly manifests in patients grappling with poorly controlled bronchial asthma and those afflicted by cystic fibrosis. This condition represents a rare inflammatory lung ailment characterized by an aberrant immune response to Aspergillus. The presence of thick mucus within the airways can impede the efficient clearance of inhaled Aspergillus spores. Furthermore, investigations have uncovered indications of genetic susceptibility, with specific single nucleotide polymorphisms (SNPs), notably TLR3, IL4R, and IL13, being linked to an elevated predisposition for ABPA [1].

## Case presentation

A 58-year-old male patient with a long-standing history of bronchial asthma presented for evaluation. His initial treatment consisted of inhaled dry powder steroid inhalers, which were subsequently transitioned to combined metered-dose inhalers. Over the 28-year duration of management, the treatment regimen exhibited inconsistencies. Upon presentation, the patient reported progressive exertional dyspnea (Modified Medical Research Council {mMRC} Dyspnea scale - grade III) persisting for the past six months [2]. This dyspnea was accompanied by a non-purulent productive cough. Additionally, he noted a loss of appetite and weight without concurrent complaints of fever, hemoptysis, night sweats, or other constitutional symptoms indicative of tuberculosis.

Clinical examination revealed the absence of fever (98.6°F), a respiratory rate of 18 breaths per minute, oxygen saturation of 92% on room air, a heart rate of 96 beats per minute, and a blood pressure of 130/80 mmHg. Jugular venous pressure (JVP) was within normal limits. Auscultation of the chest revealed vesicular breathing with reduced breath sounds equally distributed in both bilateral lung bases, along with crepitations in both lower lung zones. No finger clubbing was noted. The remainder of the physical examination yielded unremarkable findings.

Laboratory investigations disclosed a white blood cell count of 10,650/mm³ with a neutrophil predominance and an elevated eosinophil percentage of 16%. The erythrocyte sedimentation rate (ESR) was 42 mm in the first hour, and C-reactive protein (CRP) was measured at 23 mg/dL. A 2D echocardiogram was performed to rule out heart failure, while spirometry demonstrated moderate large and small airway obstruction with reversibility, consistent with the clinical picture of chronic asthma.

Microbiological investigations, including sputum analysis for acid-fast bacilli, GeneXpert for *Mycobacterium tuberculosis*, and sputum acid-fast bacillus (AFB) culture, all yielded negative results, effectively excluding pulmonary tuberculosis. Furthermore, sputum cultures for pyogenic and fungal organisms exhibited no growth.

**Figure 1:**
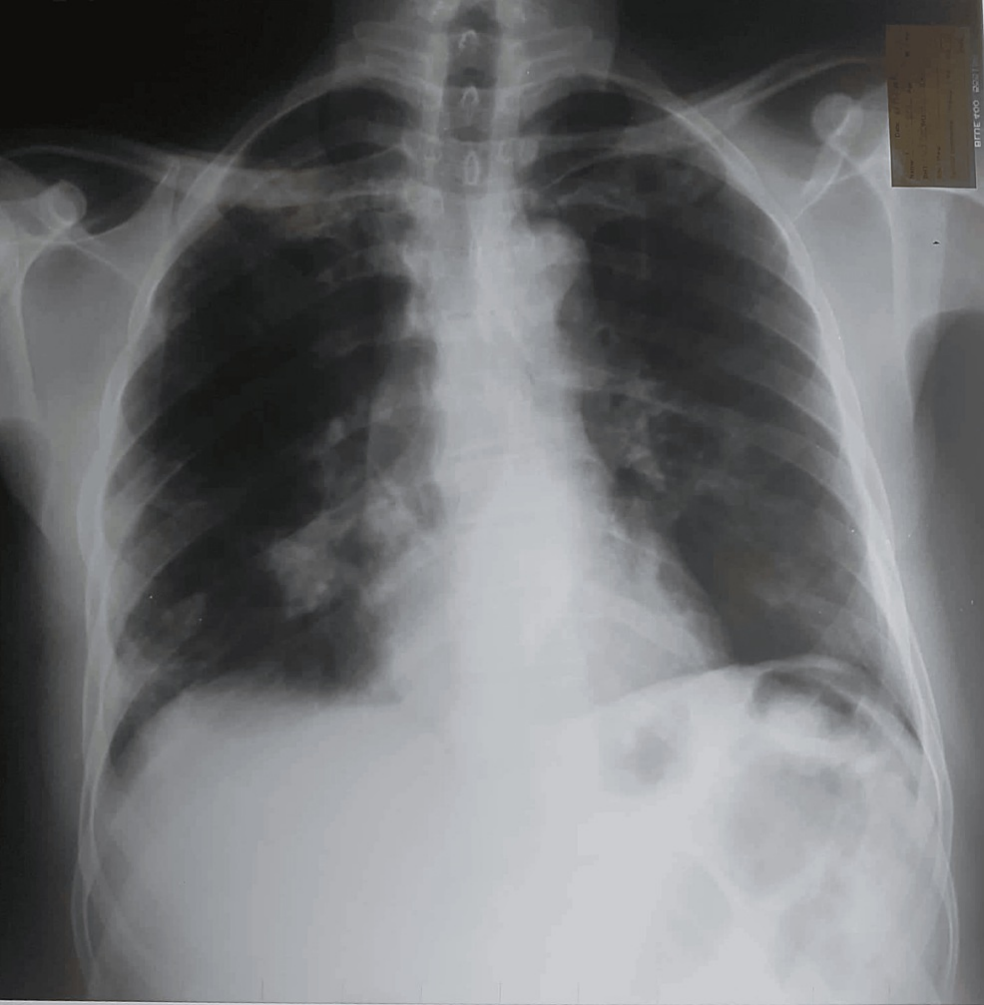
Chest x-ray showing transient pulmonary opacities in bilateral lung bases.

**Figure 2:**
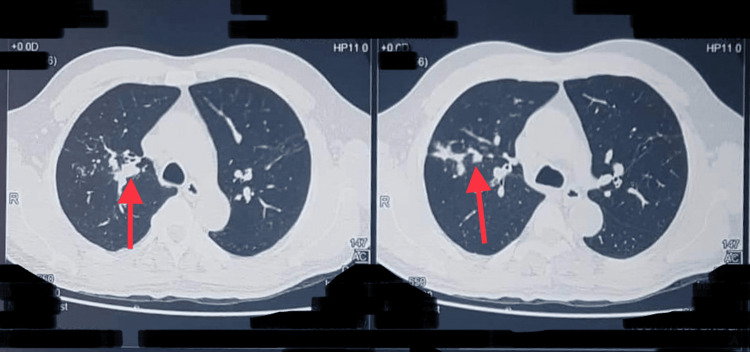
Features of proximal bronchiectasis with areas of mucus impaction.

Chest x-ray exhibited transient pulmonary opacities in bilateral lung bases, with the remaining lung fields appearing normal (Figure 1). High-resolution computed tomography (HRCT) of the chest revealed characteristic features of proximal bronchiectasis with areas of mucus impaction (Figure 2). Additionally, it was noted to have areas of tree in bud appearance suggestive of inflammation.

However, given the backdrop of poorly controlled bronchial asthma, fungal infections needed to be expeditiously ruled out. Serum assays, including Aspergillus antigen, Aspergillus-specific antibodies, and total IgE levels, were performed. These tests revealed elevated serum IgE levels of 1928 U/L, a serum Aspergillus antigen of 5.26 (normal: <0.5), and positive IgE and IgG serum Aspergillus antibodies.

Subsequently, the patient underwent bronchoalveolar lavage (BAL), revealing eosinophilic infiltration along with a sparse presence of alveolar macrophages and occasional fungal hyphae. The Aspergillus galactomannan antigen level was elevated at 4.31 (normal: <0.5), while bronchial fluid cultures showed no growth of microorganisms.

Based on the clinical history of chronic, uncontrolled bronchial asthma, peripheral blood eosinophilia, elevated serum IgE levels, and positive serum and BAL antigens for Aspergillus, alongside significantly increased serum antibodies against Aspergillus, a diagnosis of allergic bronchopulmonary aspergillosis (ABPA) was established. Radiological findings corroborated this diagnosis.

The patient's treatment regimen included oral voriconazole at a loading dose of 300 mg, followed by 200 mg twice daily for a duration of six months, along with oral prednisone at 30 mg daily, and metered dose inhaler (MDI) - fluticasone/salmeterol 250/25 mcg two puffs twice daily with a spacer device as pharmacological management [3]. The patient received comprehensive health education regarding the disease, prognosis, and preventive measures. Pneumococcal and influenza vaccines were administered, and nutritional counseling was provided as non-pharmacological management. Monthly follow-up visits were recommended, with liver function tests and serial measurements of serum IgE levels monitored to track remission progress.

## Discussion

Aspergillus species represent ubiquitous molds prevalent in organic substrates. While over 100 species have been identified, human affliction predominantly arises from *Aspergillus fumigatus *and *Aspergillus* *niger*, with infrequent instances stemming from *Aspergillus flavus *and *Aspergillus clavatus* [4]. Fungal spore transmission to the human host occurs through inhalation [5].

Aspergillus is linked to a range of diseases within the human host, encompassing hypersensitivity reactions to direct angioinvasion. The primary site of impact for Aspergillus is the pulmonary system, giving rise to four principal syndromes. These include allergic bronchopulmonary aspergillosis (ABPA), chronic necrotizing Aspergillus pneumonia (CNPA) or chronic necrotizing pulmonary aspergillosis, aspergilloma, and invasive aspergillosis [6]. These syndromes represent distinct clinical manifestations associated with Aspergillus infections in the respiratory system.

Nonetheless, in profoundly immunocompromised patients, Aspergillus can disseminate hematogenously beyond the pulmonary realm, potentially inciting endophthalmitis, endocarditis, myocardial, renal, hepatic, splenic, soft tissue, central nervous system (CNS), and osseous abscesses.

ABPA denotes a hypersensitivity retort to *Aspergillus fumigatus *colonization within the tracheobronchial tree, often co-occurring with asthma and cystic fibrosis (CF). Allergic fungal sinusitis can also manifest either in isolation or concomitantly with ABPA. Bronchocentric granulomatosis and malt worker's lung, both hypersensitivity lung disorders, are traced back to Aspergillus species, albeit they are infrequent occurrences.

An aspergilloma manifests as a fungus ball (mycetoma) evolving within a preexisting lung parenchymal cavity. Predisposing factors for cavitary disease encompass treated tuberculosis, necrotizing infections, sarcoidosis, CF, and emphysematous bullae. While the fungal ball can shift within the cavity, it refrains from infiltrating the cavity wall, although it might incite hemoptysis.

CNPA represents a subacute process typically discerned in patients displaying degrees of immunosuppression, commonly linked to underlying lung disorders, alcoholism, or extended corticosteroid usage. Given its relative rarity, CNPA frequently evades timely recognition, progressing over weeks or months, and inducing a gradual cavitary pulmonary infiltrate [7].

Invasive aspergillosis, a swiftly advancing and often lethal infection, transpires in profoundly immunosuppressed patients, prominently those profoundly neutropenic. This infectious course is hallmarked by vascular invasion, yielding multifocal infiltrates that are frequently wedge-shaped, pleural-based, and cavitary. Dissemination to other organs, particularly the CNS, can materialize.

## Conclusions

This study has provided an insightful examination of a patient afflicted by chronic, uncontrolled bronchial asthma, who presented with persistent and unresolved symptoms, notably exertional dyspnea. It underscores the utmost significance of considering the possibility of allergic bronchopulmonary aspergillosis (ABPA) in individuals grappling with chronic bronchial asthma, particularly when confronted with recurrent respiratory tract infections. The early suspicion, diagnosis, and management of ABPA in such cases are paramount. Timely intervention not only alleviates immediate symptoms but also holds the potential to mitigate the long-term morbidity associated with irreversible lung changes.

Moreover, it is imperative for the medical community to heighten awareness regarding ABPA as a potential complication in chronic bronchial asthma, fostering a proactive approach to its diagnosis and management. Enhanced understanding and vigilance regarding ABPA can facilitate early interventions, which, in turn, can ameliorate the burden of chronic respiratory morbidity in affected individuals. This study serves as a poignant reminder of the pivotal role healthcare practitioners play in recognizing and addressing ABPA within the context of chronic bronchial asthma, ultimately advancing the prospect of improved patient outcomes and overall well-being. Continued research and clinical investigations are warranted to refine diagnostic strategies and therapeutic modalities for ABPA, further enhancing the efficacy of interventions and diminishing the specter of long-term complications.
